# Mitochondrial metabolic dysfunction drives PANoptosis in retinal pigment epithelium during fungal endophthalmitis: emerging roles of the MITF–FBXW7 axis

**DOI:** 10.3389/fimmu.2026.1826337

**Published:** 2026-04-13

**Authors:** Xiaohan Zhang, Jinfeng Zhang, Xuesong Lin

**Affiliations:** Department of Ophthalmology, Ningde Municipal Hospital, Ningde Clinical Medical College of Fujian Medical University, Ningde, China

**Keywords:** fungal endophthalmitis, host-directed therapy, mitochondrial homeostasis, PANoptosis, retinal pigment epithelium, ZBP1

## Abstract

Fungal endophthalmitis (FE), although less common than bacterial endophthalmitis, carries a disproportionately high risk of irreversible blindness. Clinical observations show that some patients continue to experience progressive visual loss even after successful microbiological clearance, suggesting that disease outcomes are strongly influenced by excessive host immune−inflammatory injury rather than pathogen burden alone. Focusing on the retinal pigment epithelium (RPE), a key component of the blood–retinal barrier, this review summarizes recent advances in intraocular microenvironmental alterations, RPE immune responses, and the remodeling of cell death pathways during FE pathogenesis. We outline a conceptual framework centered on a “metabolism–immunity–death” axis. In this model, fungal infection induces mitochondrial metabolic reprogramming and dynamic imbalance in RPE cells, which can be associated with cytosolic leakage of mitochondrial DNA (mtDNA). As a danger−associated molecular pattern, mtDNA may activate the Z−DNA binding protein 1 (ZBP1) sensor, promote PANoptosome assembly and coordinate inflammatory cell death programs including pyroptosis, apoptosis, and necroptosis. We further highlight the regulatory GSK3β–MITF–FBXW7 axis and discuss how its dysregulation may connect impaired metabolic adaptation with irreversible RPE PANoptosis. Finally, potential translational implications of host−directed therapy (HDT) are discussed, including the use of cell−free mtDNA as an early biomarker and therapeutic strategies that combine metabolic protection with antifungal treatment. Collectively, this review provides a mechanistic perspective on the poor visual outcomes of FE and identifies potential targets for retinoprotective intervention.

## 1 Introduction

Fungal endophthalmitis (FE) is a severe ocular infection that often results in irreversible retinal damage and substantial vision loss. Despite advances in pathogen elimination through pars plana vitrectomy and antifungal pharmacotherapy, many patients continue to experience poor visual outcomes even after the infection has been controlled (1–3). This clinical paradox indicates that pathogen clearance alone does not necessarily halt tissue injury. Increasing evidence suggests that excessive host immune−inflammatory responses, together with disruption of the blood−retinal barrier (BRB), serve as major drivers of retinal structural damage (3, 4). The retinal pigment epithelium (RPE) forms a critical defense line that maintains BRB integrity, and the survival of RPE cells strongly influences visual outcomes following infection (5, 6). However, the classical view that retinal injury is primarily mediated by apoptosis cannot fully explain the intense inflammatory responses and tissue necrosis frequently observed in FE. Recent studies indicate that cell death in infectious microenvironments often involves the coordinated activation of multiple programmed death pathways, including pyroptosis, apoptosis, and necroptosis. This integrated form of inflammatory cell death has been termed PANoptosis (7, 8). Beyond causing cell loss, PANoptosis also promotes the release of inflammatory mediators that amplify tissue damage. Mitochondria are central regulators of both cellular metabolism and innate immune signaling. Mitochondrial dysfunction and the abnormal release of mitochondrial DNA (mtDNA) are increasingly recognized as key danger signals that activate inflammatory cell death pathways (9). From this perspective, mitochondrial homeostasis may represent an important upstream determinant of RPE survival during infection.

In this review, we examine how mitochondrial metabolic reprogramming may promote RPE PANoptosis through innate immune sensors such as Z−DNA binding protein 1 (ZBP1). We also discuss the role of the glycogen synthase kinase−3β (GSK3β)–microphthalmia−associated transcription factor (MITF)–F−box/WD repeat−containing protein 7 (FBXW7) regulatory axis in mitochondrial biogenesis and quality control, and how disruption of this pathway may contribute to mtDNA leakage and downstream inflammatory cell death. By integrating recent findings from immunometabolism and retinal biology, this review aims to provide a theoretical basis for host−directed therapeutic strategies targeting mitochondrial homeostasis to improve visual outcomes in FE.

To provide an overview of current research in this area and support the proposed framework, a literature search was performed using the PubMed, Web of Science, and Scopus databases. The search focused primarily on English−language articles published within the past decade. Keywords included, but were not limited to, “fungal endophthalmitis,” “retinal pigment epithelium,” “mitochondrial dysfunction,” “PANoptosis,” “ZBP1,” and “FBXW7/MITF.” After removing duplicate records and studies with limited relevance, experimental studies and high−quality reviews related to ocular tissues, immunometabolism, and inflammatory signaling were selected. This approach was intended to ensure that the conceptual framework proposed in this review is supported by available experimental and mechanistic evidence.

## 2 Clinical characteristics and therapeutic limitations of FE

### 2.1 Epidemiological features and pathogenic evolution

Historically, FE mostly occurred secondary to penetrating ocular trauma or intraocular surgery, classifying it primarily as an exogenous infection. Over the past two decades, however, epidemiological data indicate a rising incidence of endogenous FE (EFE), a trend closely tied to the prevalence of systemic fungemia (9). Intravenous fluid therapy in primary or rural healthcare settings is emerging as an under-discussed risk factor. This hematogenous dissemination route not only increases disease risk but diversifies the spectrum of infecting pathogens (10–14). Differences in fungal species strongly influence clinical manifestations and disease severity. A pooled analysis by Chen et al. (15) showed that *Candida* species remain the most common causative pathogens, accounting for more than half of reported cases. These infections often present as relatively localized chorioretinal lesions and may have favorable outcomes when treated promptly. In contrast, filamentous fungi such as *Aspergillus* exhibit stronger tissue invasiveness. These pathogens can rapidly induce retinal vascular occlusion and extensive necrosis, leading to more severe visual damage (2). In addition, the intraocular environment may exert selective pressure on invading fungi, promoting adaptive responses that facilitate persistence. Such adaptations include dense biofilm formation that reduces drug penetration and structural modification of the fungal cell wall to evade host immune recognition (16). These changes may compromise the effectiveness of empirical antifungal therapy and delay timely treatment, ultimately reducing ocular salvage rates (17).

### 2.2 Intraocular microenvironmental alterations and loss of immune privilege

**Figure 1:**
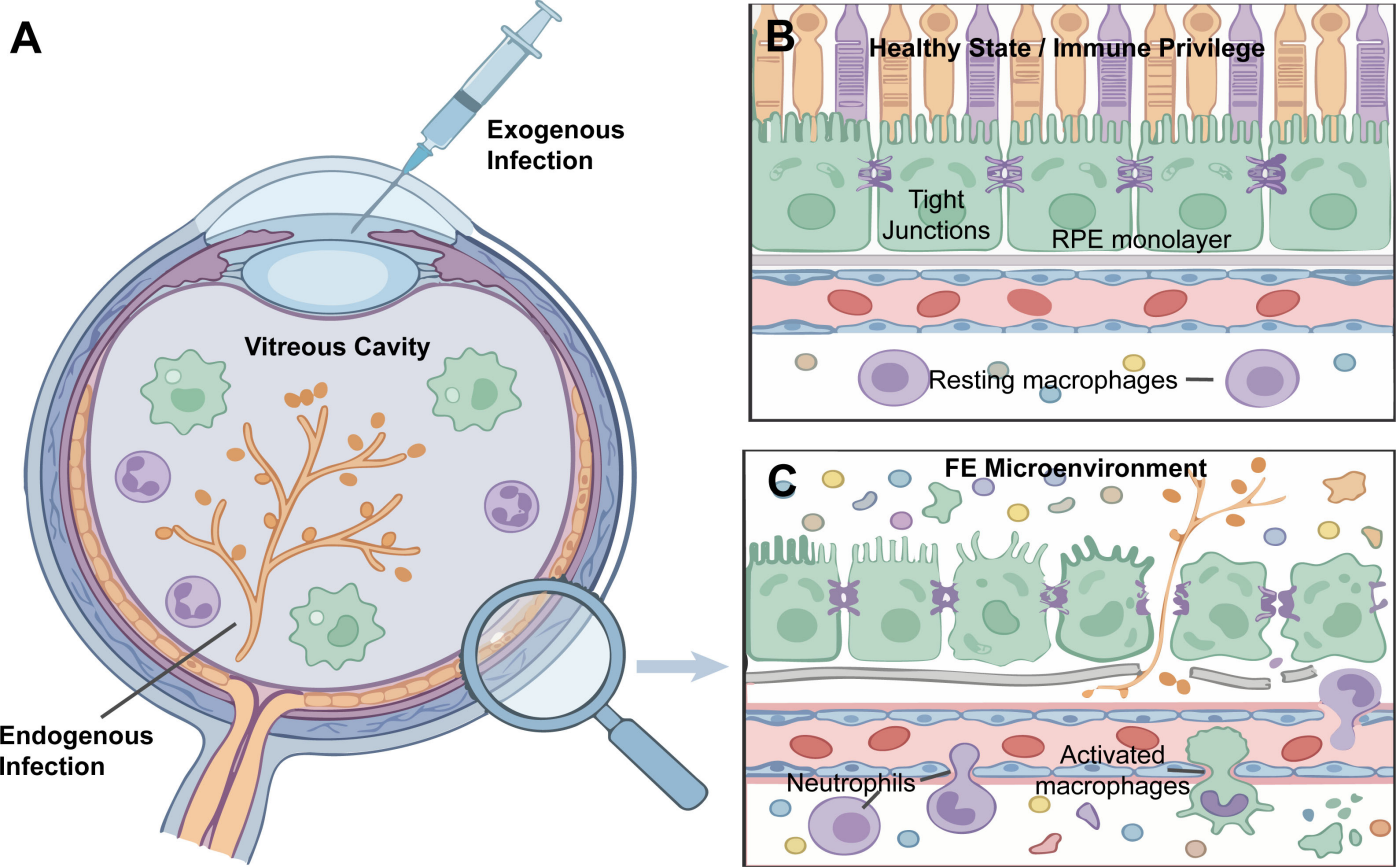
Routes of ocular fungal infection and breakdown of the outer blood-retinal barrier (BRB). **(A)** Schematic overview of the two primary routes of ocular fungal infection: exogenous and endogenous. Fungal elements proliferate within the vitreous cavity. **(B)** The healthy state representing an immune-privileged microenvironment, maintained by an intact RPE monolayer and tight junctions. **(C)** The Fungal Endophthalmitis (FE) microenvironment. Fungal invasion disrupts tight junctions and triggers a massive infiltration of innate immune cells, particularly neutrophils and activated macrophages, amplifying localized inflammation.

The eye is considered a classical immune-privileged organ, relying on an intact BRB and a sustained immunosuppressive microenvironment for homeostasis (18). Fungal infection rapidly abolishes this delicate balance (**Figure 1**). Once fungal cell wall components (particularly β-glucans)—acting as typical pathogen-associated molecular patterns (PAMPs)—breach the physical barrier and enter the eye, they trigger innate immune recognition, sparking a profound inflammatory response characterized by neutrophil infiltration (19). The unique anatomical and physicochemical properties of the vitreous cavity further complicate treatment. Its gel−like structure can restrict the effective intraocular distribution of systemically administered drugs while simultaneously providing a relatively favorable niche for pathogen persistence. Fungal aggregates within the vitreous may enter a low−metabolic state, which reduces their susceptibility to antifungal agents that primarily target actively growing cells. At the same time, the continuous diffusion of fungal virulence factors and inflammatory mediators amplifies tissue injury, eventually affecting retinal ganglion cells and contributing to irreversible visual impairment (5).

### 2.3 Pathophysiological role of RPE cells in fungal infection

RPE cells form the central structural component of the outer BRB and play an essential role in maintaining retinal homeostasis (20). During FE, RPE cells are not merely passive targets of infection but also function as active participants in immune defense. Studies have shown that RPE cells express multiple pattern recognition receptors, including TLR2/4 and Dectin−1, which allow them to directly detect fungal components and initiate downstream immune responses (19). Upon infectious stimulation, the barrier properties of RPE cells are compromised and their phenotype shifts toward a pro−inflammatory state. Under these conditions, RPE cells release chemokines such as IL−8 and MCP−1 that recruit inflammatory cells to the site of infection (21). While this response contributes to pathogen clearance, RPE injury is not driven solely by external inflammatory signals. Disruption of intracellular metabolic homeostasis also plays an important role. Emerging evidence suggests that RPE cells undergo mitochondrial metabolic reprogramming during acute infection to meet increased energy demands (22, 23). Although this metabolic adaptation may temporarily support immune responses, prolonged stress can lead to oxidative damage and mitochondrial dysfunction, ultimately impairing RPE metabolism (24). The accumulation of such damage may trigger programmed cell death pathways. Recent multi−omics studies in ophthalmology further indicate that under sustained inflammatory stress and microenvironmental imbalance—such as in retinal degenerative diseases including age−related macular degeneration—progressive RPE loss may involve a highly inflammatory PANoptotic program rather than a single cell−death pathway (25). Extensive RPE degeneration leads to irreversible breakdown of the outer BRB and is strongly associated with poor visual outcomes in FE patients (15).

### 2.4 Current therapeutic limitations and the rationale for host-directed therapy

At present, intravitreal administration of antifungal agents such as voriconazole or amphotericin B remains the cornerstone of FE treatment. However, several limitations restrict the effectiveness of this approach in clinical practice. Many antifungal agents have relatively short intraocular half−lives, which often necessitates repeated injections and increases the risk of complications, including intraocular hemorrhage and retinal detachment. In addition, the narrow therapeutic window of certain drugs means that small deviations in dosage may lead to retinal toxicity (19, 26–29). Riddell et al. (26) noted that even when new antifungal compounds demonstrate strong activity *in vitro*, achieving adequate intraocular concentrations remains challenging because of limited penetration across the BRB. More importantly, current therapeutic strategies primarily focus on pathogen eradication and provide limited protection against infection−induced immune−inflammatory injury.

The use of corticosteroids as adjunctive therapy remains controversial. Although they may suppress inflammation, their immunosuppressive effects could potentially impair antifungal defense and increase the risk of pathogen dissemination (2). In this context, HDT has been proposed as a complementary strategy for FE management (17). Alongside effective antifungal treatment, therapeutic interventions aimed at restoring mitochondrial quality control in RPE cells and limiting mtDNA−mediated inflammatory signaling may help increase cellular tolerance to inflammatory stress and reduce PANoptosis. Such approaches may represent an important direction for overcoming current therapeutic limitations and improving visual outcomes in FE patients (30).

## 3 Remodeling of cell death modalities

### 3.1 Limitations of classical death pathways and the failure of monotargeted interventions

**Figure 2:**
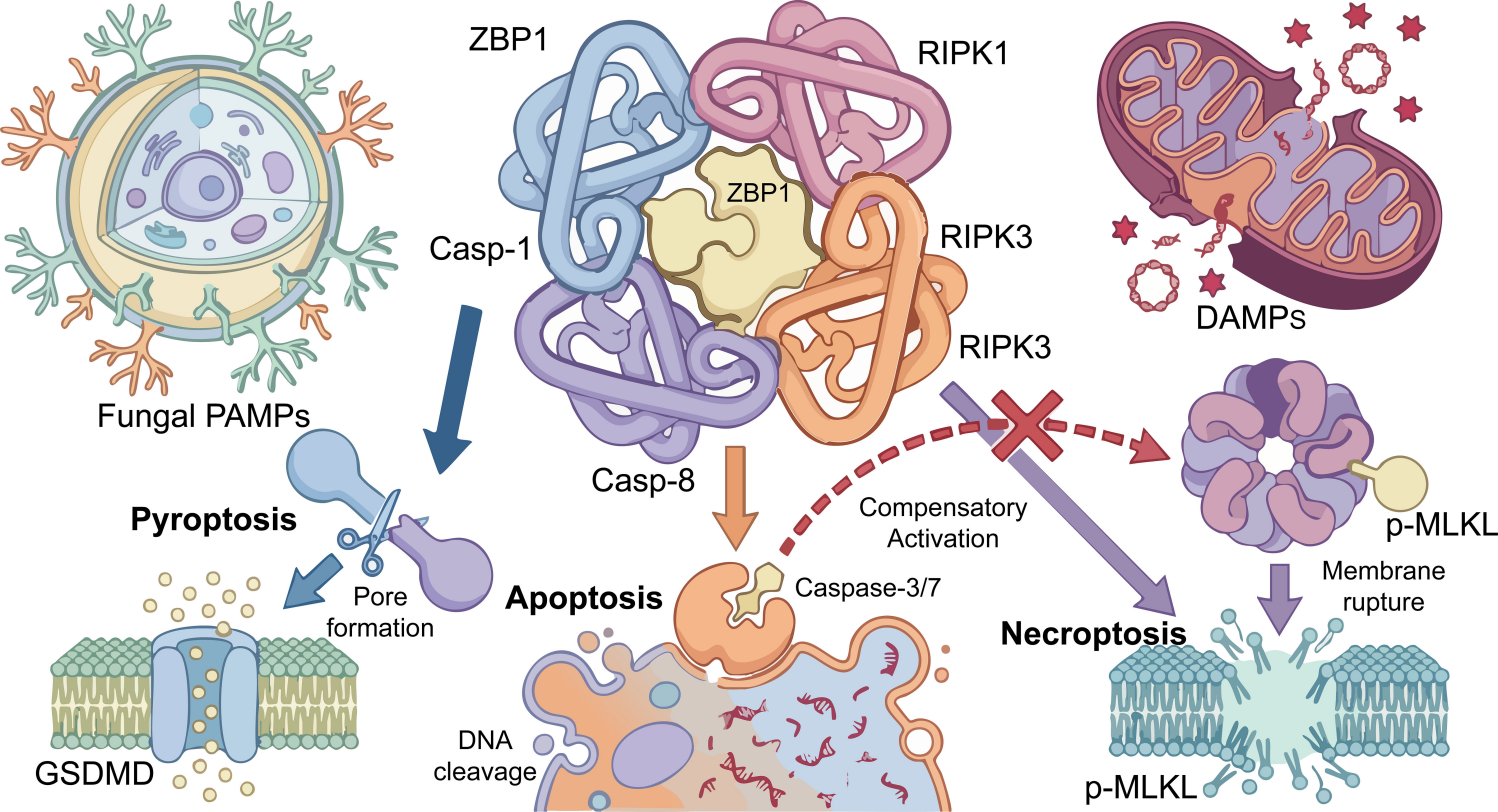
Schematic model of PANoptosome assembly and compensatory cell death mechanisms during fungal infection. Following the internalization of fungal PAMPs, cells assemble the PANoptosome complex, utilizing ZBP1 as a central scaffold to recruit key regulatory kinases (RIPK1, RIPK3) and caspases (Caspase-1, Caspase-8). This interaction drives the synchronous execution of pyroptosis, apoptosis, and necroptosis. Notably, isolated inhibition of the apoptotic pathway fails to prevent cell death; instead, it provokes a compensatory activation of necroptosis (indicated by the red pathway). This mechanistic shift exacerbates cell membrane rupture and the massive release of DAMPs, ultimately amplifying the inflammatory storm within the retinal microenvironment.

For many years, studies of infection−induced RPE injury primarily focused on classical apoptosis. Previous efforts aimed to preserve retinal function by selectively inhibiting Caspase−3 or Caspase−8; however, these strategies generally failed to achieve the expected therapeutic effects in FE models. Growing evidence indicates that fungal infection can induce multiple non−apoptotic forms of cell death. For example, *Aspergillus* infection activates NLRP3 inflammasome−mediated pyroptosis, whereas certain mycotoxins trigger RIPK1/3−dependent necroptosis. Indeed, fungal pathogens like Aspergillus fumigatus and Candida albicans have been shown to induce hallmarks of PANoptosis, including inflammasome activation and cell death, typically within 6 hours post-infection in macrophage models (7). This diversity of death pathways suggests that targeting a single mechanism is unlikely to be sufficient. Experimental observations further show that inhibition of apoptosis alone often leads to compensatory activation of necroptosis (illustrated by the red compensatory pathway in **Figure 2**). Unlike apoptosis, necroptosis leads to membrane rupture and the release of large amounts of damage−associated molecular patterns (DAMPs), thereby amplifying the local inflammatory response (31–33). These findings indicate that cell death in the infectious microenvironment operates as an interconnected network rather than as isolated pathways. Consequently, it is necessary to move beyond single−pathway models and focus on upstream regulatory mechanisms capable of coordinating multiple death programs.

### 3.2 Molecular characteristics and synergistic activation mechanisms of the PANoptotic pathway

In 2019, Malireddi et al. introduced the concept of “PANoptosis”, which provides a unified framework for understanding infection−associated cell death (8, 34). It is crucial to clarify that this process is not simply a coexistence of multiple death pathways, nor is it merely the parallel activation of pyroptosis, apoptosis, and necroptosis. Instead, true PANoptosis requires the coordinated activation and physical integration of key molecular components of these pathways within the same cell into a unified multiprotein complex known as the PANoptosome, typically in response to specific pathogens or stress signals (35, 36). The central feature of PANoptosis is the formation of a multiprotein complex known as the PANoptosome. Sensor molecules such as ZBP1 often serve as scaffolds for this complex, recruiting Caspase−1 (a pyroptotic effector), Caspase−8 (an apoptotic initiator), and RIPK3 (a key necroptotic kinase) through protein–protein interactions (8, 36) (see the PANoptosome assembly model in **Figure 2**). Crucially, this scaffold facilitates the intricate molecular integration and functional convergence of these pathways. For instance, within the PANoptosome, inflammasome activation leads to the maturation and activation of Caspase-1, which subsequently cleaves Gasdermin D (GSDMD) to initiate pyroptosis. Concurrently, the RIPK1/RIPK3 kinase axis, central to necroptosis, is also recruited and activated. This represents a molecular crosstalk where inflammasome-mediated Caspase-1 activity and RIPK signaling are not isolated but rather interlinked and mutually influenced within the same complex. Through this coordinated activation, infected host cells can be eliminated efficiently.

Despite this conceptual framework, direct studies definitively demonstrating PANoptosome formation and true PANoptosis in RPE cells under infectious conditions remain limited. Much of the current evidence, while showing upregulation of markers from multiple cell death pathways, often points to multi-modal cell death rather than confirmed PANoptotic integration. Nevertheless, recent retinal single−cell transcriptomic analyses have demonstrated that abnormal activation of the PANoptotic network is a prominent feature of immune dysregulation and degenerative injury within the RPE microenvironment (25). When these observations are considered together with evidence that fungal pathogens can induce ZBP1−dependent PANoptosis in immune cell models (7, 37), as well as reports highlighting the involvement of ZBP1 in retinal degeneration (38), a plausible pathological hypothesis emerges. In FE, excessive activation of PANoptosis in RPE cells may represent a potential mechanism contributing to the disruption of BRB integrity. However, further studies are essential to confirm the precise mechanisms of PANoptosome assembly and integrated cell death in RPE cells during fungal endophthalmitis.

### 3.3 ZBP1-mediated cytosolic mtDNA sensing mechanisms

During PANoptosome assembly, ZBP1 functions as the central sensor. Unlike TLR4, which recognizes bacterial lipopolysaccharide, ZBP1 specifically identifies left-handed helical conformations of Z-DNA or Z-RNA (39, 40). Previous studies have identified ZBP1 as a key mediator that is implicated in PANoptosis in host cells during fungal infections, including those caused by *Candida* and *Aspergillus* species (7). Importantly, the ligands detected by ZBP1 are not limited to pathogen−derived nucleic acids. Mitochondrial stress can lead to the release of mtDNA into the cytosol, where it functions as a potent DAMP capable of activating innate immune signaling pathways such as cGAS−STING and the NLRP3 inflammasome (41–45). Evidence from glaucoma models further indicates that mitochondrial dynamic imbalance induced by elevated intraocular pressure (for example, Drp1−mediated mitochondrial fragmentation) can directly trigger PANoptosis in retinal ganglion cells (23). In addition, recent mechanistic studies in RPE cells have shown that severe mitochondrial structural and functional damage—such as mitochondrial calcium overload resulting from abnormal endoplasmic reticulum–mitochondria tethering—can activate inflammasome signaling and induce RIPK1−dependent PANoptosis (46). These findings establish a mechanistic link between mitochondrial homeostasis and PANoptotic cell death.

Given the established role of cytosolic mtDNA in activating classical nucleic acid sensors such as cGAS−STING, two potential models may explain how mtDNA contributes to ZBP1−related PANoptosis in RPE cells during FE.

Model A *(Direct Sensing Hypothesis):* Under conditions of intense oxidative stress, highly oxidized mtDNA may undergo structural alterations that promote the formation of Z−DNA−like conformations. These structural changes could allow mtDNA to be directly recognized by ZBP1. This possibility remains largely hypothetical and requires further validation through structural and biochemical studies.

Model B *(Cascade Amplification Hypothesis):* In this model, leaked mtDNA is first detected by the classical cGAS−STING pathway, which induces strong type I interferon (IFN) responses. Elevated IFN signaling subsequently upregulates the otherwise low basal expression of ZBP1 in RPE cells through autocrine or paracrine mechanisms. Increased ZBP1 expression may then sensitize cells to fungal toxins or additional DAMP signals, ultimately contributing to PANoptosome assembly.

Regardless of which mechanism predominates, cytosolic mtDNA leakage likely functions as an important catalytic signal in this pathogenic cascade. This framework also provides a possible explanation for a clinical observation in FE: even after antifungal therapy successfully eliminates the pathogen, inflammatory injury and cell death in RPE tissue may persist if mitochondrial function has not been restored.

## 4 Mitochondrial metabolic reprogramming and RPE PANoptosis

### 4.1 Central role of mitochondria in immunometabolic regulation

RPE cells face substantial physiological demands, including phagocytosis of photoreceptor outer segments and regulation of transmembrane ion transport, processes that require high levels of ATP production (6, 47, 48). In the pathological microenvironment of FE, mitochondrial function extends beyond energy generation and undergoes marked adaptive changes. Studies in immunometabolism suggest that mitochondria serve as key hubs integrating pathogen−derived signals with host metabolic status (22). Under sustained stimulation by inflammatory cytokines, the functional role of RPE mitochondria often shifts from efficient ATP production toward immune and stress signaling. This transition is closely associated with downstream programs of inflammatory cell death. Such metabolic reprogramming frequently precedes visible morphological changes and may represent a critical point determining whether RPE cells adapt to stress or progress toward irreversible injury (49). This immunometabolic shift, profoundly impacting RPE cell fate, is subject to intricate cellular regulatory mechanisms, including those potentially involving the GSK3β–MITF–FBXW7 axis.

### 4.2 Mitochondrial dynamic imbalance and mtDNA leakage

Normal mitochondrial function depends on a dynamic balance between fusion and fission. Under the intense oxidative stress associated with fungal infection, this balance becomes highly vulnerable to disruption. Previous studies have shown that the mitochondrial fission regulator Drp1 becomes aberrantly activated and phosphorylated, promoting excessive mitochondrial fragmentation (23, 50). GSK3β has been identified as an important upstream kinase involved in the regulation of Drp1 activity. Ophthalmic studies have reported that abnormal activation of GSK3β increases Drp1 expression and promotes its translocation to mitochondria, thereby facilitating pathological mitochondrial hyper−fission (51). Severe mitochondrial fragmentation not only increases membrane permeability and causes a loss of mitochondrial membrane potential but also promotes the release of mtDNA into the cytosol. Once released, mtDNA can be detected by intracellular DNA sensors such as cGAS−STING or NLRP3, which recognize it as a danger signal (41, 52, 53). Activation of these pathways may increase inflammatory signaling and contribute to PANoptosome formation. In this context, disruption of mitochondrial dynamics may represent a structural basis underlying the initiation of PANoptosis.

### 4.3 Modification of the PANoptotic complex by reactive oxygen species accumulation

During the early stage of infection, RPE cells produce moderate levels of reactive oxygen species (ROS) through respiratory burst mechanisms that help eliminate fungal pathogens. However, under conditions of sustained intraocular inflammation, excessive ROS accumulation becomes a source of oxidative damage. One contributing factor is the impairment of endogenous antioxidant defenses and mitochondrial quality control systems. The transcription factor MITF, which plays an important role in oxidative stress regulation, has been reported to be significantly downregulated during persistent cellular stress. Reduced MITF activity weakens antioxidant responses and also disrupts mitochondrial integrity by decreasing transcription of mitochondrial fusion proteins such as MFN2. As a result, mitochondrial stability declines and ROS leakage increases (54). Elevated ROS levels can induce lipid peroxidation and compromise cellular membrane integrity. In addition, ROS further destabilizes mitochondrial membranes, facilitating mtDNA release and serving as an important signal that may promote activation of the NLRP3 inflammasome (43, 55–57). This mechanism may explain why treatment strategies relying solely on conventional antioxidants, such as vitamin C supplementation, often show limited effectiveness; simply scavenging ROS does not reverse the underlying mitochondrial structural damage or the downstream death pathways that have already been initiated.

### 4.4 Glycolytic shift and the disruption of cellular energy homeostasis

During acute infection, RPE cells may undergo metabolic changes similar to the “Warburg effect” observed in tumor cells (58, 59). In this state, cells shift from efficient oxidative phosphorylation (OXPHOS) toward a faster but less efficient glycolytic pathway to meet the biosynthetic demands associated with immune activation, including cytokine production. Although this metabolic adjustment may support short−term cellular responses, prolonged reliance on glycolysis leads to lactate accumulation and acidification of the local microenvironment, which can ultimately contribute to metabolic stress and energy imbalance (60). When intracellular ATP levels fall below the threshold required for maintaining cellular homeostasis, ion pump activity fails, which is associated with cell swelling and membrane disruption. Therefore, pharmacological approaches that restore mitochondrial function and promote a shift back toward normal OXPHOS metabolism may represent a potential strategy for limiting PANoptotic cell death in RPE cells (61).

Given that mitochondrial metabolic disturbance and dynamic imbalance appear to play central roles in RPE PANoptosis, identifying upstream regulators capable of coordinating mitochondrial biogenesis together with fusion–fission balance is thus essential for interrupting this pathogenic cycle.

## 5 Role of the MITF/FBXW7 signaling axis in maintaining metabolic homeostasis and regulating PANoptosis

### 5.1 MITF as a key regulator of RPE mitochondrial biogenesis

MITF has traditionally been considered a central regulator of RPE differentiation and pigment production. More recent studies have revealed additional roles for MITF in cellular metabolic regulation (62). In particular, MITF has been identified as an upstream regulator of peroxisome proliferator−activated receptor−gamma coactivator 1−alpha (PGC−1α), a core factor controlling mitochondrial biogenesis. In RPE cells, maintenance of basal PGC−1α expression by MITF supports the continuous generation of new mitochondria, which helps replace damaged organelles and maintain mitochondrial network integrity (63). Evidence further indicates that MITF can directly bind to the promoter region of mitofusin 2 (MFN2), thereby promoting mitochondrial fusion and limiting oxidative stress–induced injury (54). Adequate MITF activity therefore helps RPE cells meet high metabolic demands while keeping ROS accumulation and cytosolic mtDNA release within physiological limits, thereby potentially restraining activation of the PANoptotic pathway at an early stage (43). Overall, the GSK3β–MITF–FBXW7 axis is proposed to function as an immunometabolic link between mitochondrial homeostasis and inflammatory priming, providing a mechanistic basis for its involvement in RPE PANoptotic dysregulation.

### 5.2 Dynamic regulation of transcription factors by the ubiquitin-proteasome system

During acute stress triggered by fungal infection, cells frequently rely on the ubiquitin−proteasome system (UPS) to selectively degrade proteins and rapidly adjust gene expression programs. FBXW7 plays an important role in this process. As a substrate−specific E3 ubiquitin ligase, FBXW7 was initially characterized for its ability to target oncogenic proteins such as c−Myc and Notch for degradation in tumor cells. More recently, studies have begun to highlight its functions in terminally differentiated and non−proliferating cells, including RPE cells, particularly in the context of stress−induced regulation of transcription factors. These observations suggest that FBXW7 may function not only as a tumor suppressor but also as a regulatory node that is involved in cellular stress responses.

### 5.3 FBXW7-mediated, phosphorylation-dependent degradation of MITF

Molecular studies indicate that recognition of MITF by FBXW7 depends on prior phosphorylation. Within the inflammatory environment associated with FE, GSK3β is often aberrantly activated. Activated GSK3β phosphorylates conserved serine residues on MITF (for example S298), creating a phospho−degron motif (64, 65). This phosphorylated motif can then be recognized by FBXW7, which promotes polyubiquitination of MITF and directs it to proteasomal degradation. The downstream consequence may include a reduced mitochondrial protective capacity in RPE cells. As mitochondrial integrity becomes compromised, mtDNA may escape into the cytosol and function as a DAMP (43, 44). Recent studies further suggest that ZBP1 may not only detect exogenous viral nucleic acids but also sense endogenous stress signals associated with abnormal mtDNA accumulation. Through this mechanism, ZBP1 may contribute to PANoptosome assembly and potentially promote activation of PANoptosis under certain stress conditions (66).

**Figure 3:**
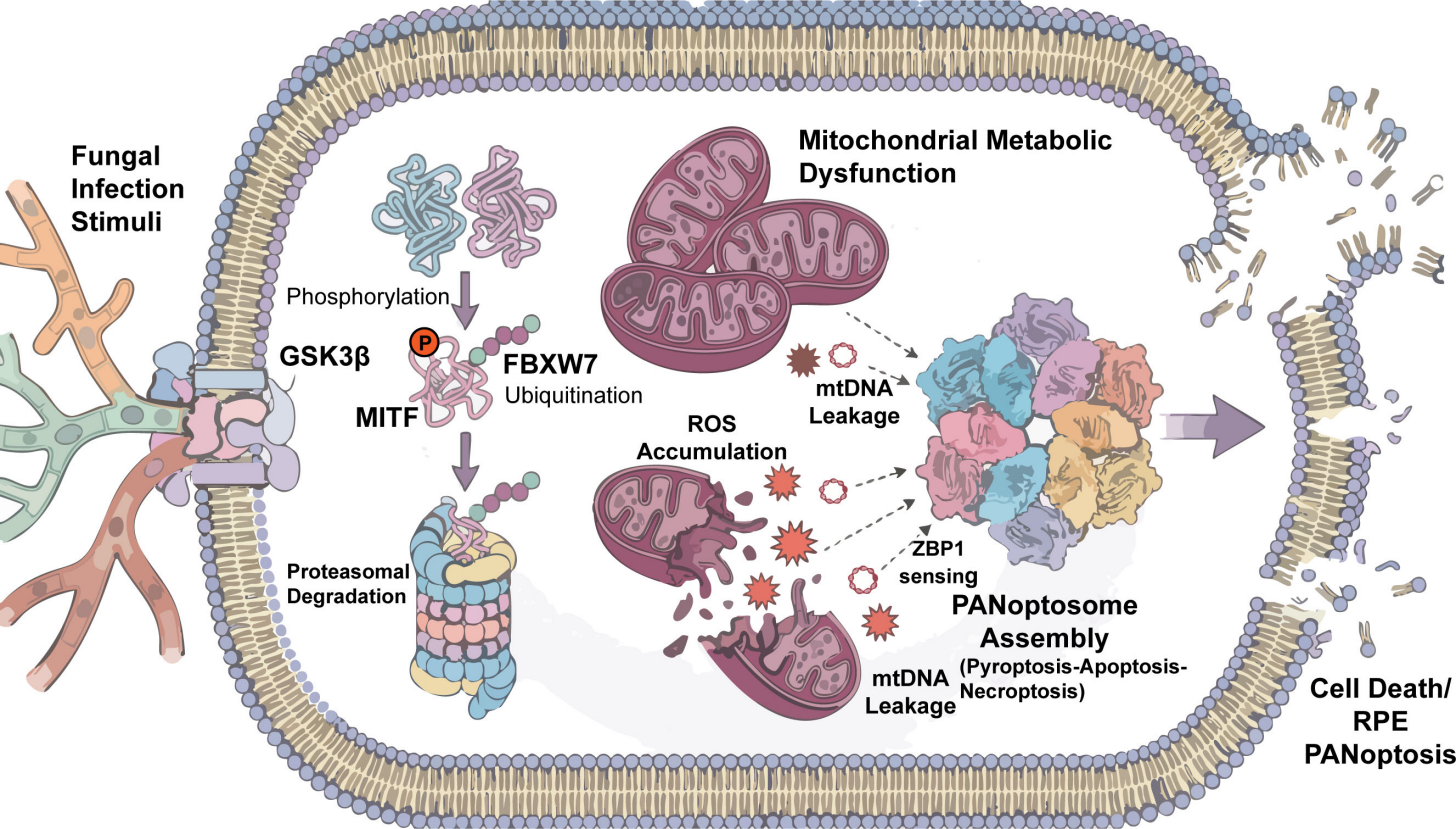
Proposed mechanism of fungal−induced RPE PANoptosis via the GSK3β–FBXW7–MITF axis. In this model, fungal infection triggers the activation of GSK3β, which phosphorylates the transcription factor MITF. This modification promotes recognition of MITF by the E3 ubiquitin ligase FBXW7, leading to its ubiquitination and subsequent proteasomal degradation. Because MITF plays an important role in maintaining mitochondrial integrity and metabolic homeostasis, its loss results in mitochondrial dysfunction. Damaged mitochondria accumulate reactive oxygen species (ROS) and release endogenous mitochondrial DNA (mtDNA) into the cytosol. The leaked mtDNA may function as an intracellular danger signal that can be sensed by ZBP1, thereby promoting PANoptosome assembly. Activation of this multiprotein complex ultimately drives RPE PANoptosis and membrane rupture.

Based on the mechanisms summarized above, the GSK3β–MITF–FBXW7 regulatory axis has been described in several cellular stress contexts, including melanoma. However, its sequential role within the fungal−infected RPE microenvironment remains largely unexplored. To organize the available evidence and identify current gaps in ocular research, we summarized and evaluated the supporting data for each proposed pathological step (**Table 1**). Building on this framework, we propose a working model for RPE injury in FE. In this model, fungal infection initiates inflammatory signaling that activates GSK3β. Activated GSK3β promotes phosphorylation−dependent destabilization of MITF and facilitates its degradation through FBXW7. Loss of MITF activity disrupts mitochondrial biogenesis and quality control, leading to accumulation of dysfunctional mitochondria and release of mtDNA. Cytosolic mtDNA may subsequently function as a danger signal sensed by innate immune receptors such as ZBP1, ultimately contributing to PANoptotic cell death (**Figure 3**). The precise contribution of this signaling axis to FE pathogenesis will require further validation through future *in vitro* and *in vivo* studies.

**Table 1:** Proposed mechanism vidence in RPE/ocular context supporting evidence from other systems (e. g., macrophages, tumors) key references.

Proposed mechanism	Evidence in RPE/Ocular context	Supporting evidence from other systems (e. g., macrophages, tumors)	Key references
Fungal breakthrough of the RPE barrier	High. Experimental studies demonstrate that *Candida albicans* can cross the outer blood–retinal barrier in mice. RPE cells respond to fungal challenge by releasing extracellular vesicles and pro-inflammatory cytokines.	High. Systemic infection models consistently show fungal dissemination and deep tissue invasion following hematogenous spread.	(19, 21)
Mitochondrial dysfunction and ROS accumulation	Moderate. Mitochondrial oxidative stress and metabolic disruption are well documented in RPE cells under pathological conditions such as AMD and glaucoma, although direct evidence in FE remains limited.	High. Mitochondrial stress is widely recognized as a central driver of inflammatory signaling and programmed cell death in systemic inflammatory diseases.	(23, 24, 46, 60)
mtDNA–cGAS–STING activation	Moderate. cGAS–STING signaling and inflammasome activation have been reported in degenerative RPE disorders such as AMD and may contribute to inflammatory signaling in retinal microenvironments.	High. Cytosolic mtDNA release is a well-established trigger of innate immune activation through the cGAS–STING pathway in multiple cell types.	(38, 41, 43, 57)
ZBP1-mediated PANoptosis	Low (Hypothesis). PANoptosis-related molecular networks have been detected in retinal degenerative diseases, but direct evidence of fungal-induced ZBP1 activation in RPE cells is currently lacking.	High. ZBP1 functions as an innate immune sensor of Z-nucleic acids and promotes pathogen-induced PANoptosis in immune cells.	(7, 25, 39, 40, 66)
MITF regulation by FBXW7	Moderate. MITF regulates mitochondrial fusion and homeostasis in RPE cells through MFN2-dependent pathways, although regulation by FBXW7 in RPE remains largely inferred.	High. FBXW7-mediated ubiquitin-proteasome degradation of MITF is well characterized and highly conserved in melanoma and other cell types.	(54, 64, 65)
Metabolic reprogramming	Moderate. Metabolic alterations in retinal tissues and glycolytic shifts in RPE cells under stress have been reported, and imaging studies suggest hypermetabolic activity in infectious retinal lesions.	High. Immunometabolic reprogramming, including Warburg-like glycolytic shifts, is widely recognized as a key regulator of immune responses and cell fate decisions.	(22, 48, 58, 59)

RPE, retinal pigment epithelium; BRB, blood–retinal barrier; FE, fungal endophthalmitis; AMD, age-related macular degeneration.

## 6 Conclusions and perspectives

### 6.1 Conclusions

Vision loss associated with FE is not simply the result of direct pathogen invasion but reflects a multifactorial pathological process involving disruption of metabolic homeostasis and dysregulated immune−inflammatory responses. This review summarizes recent progress in understanding infection−associated cell death and outlines a mechanistic framework for RPE injury centered on mitochondrial metabolic regulation. We propose that acute metabolic reprogramming and oxidative stress induced by fungal infection may contribute to FBXW7−dependent ubiquitination and degradation of the transcription factor MITF (64), and that loss of MITF activity subsequently disturbs the balance between mitochondrial biogenesis and quality control (50). This disturbance may contribute to progressive mitochondrial dysfunction, and once it exceeds the cellular tolerance threshold, irreversible RPE injury and retinal damage may occur.

### 6.2 Core hypothesis and mechanistic model

Based on currently available evidence, we propose a working hypothesis in which the GSK3β–MITF–FBXW7 axis participates in regulating PANoptosis in RPE cells, particularly during the acute phase of fungal infection and early progression of RPE injury. This regulatory axis, through its components, appears to modulate both mitochondrial metabolism and inflammatory signaling, thereby influencing the overall immunometabolic status of RPE cells. In this model, dysregulation of the GSK3β–MITF–FBXW7 axis is hypothesized to contribute to impaired mitochondrial function and metabolic reprogramming. This mitochondrial damage, which can develop within hours to days of metabolic disruption, is then associated with cytosolic release of danger molecules such as mtDNA, which may function as endogenous signals capable of activating the ZBP1−associated inflammatory cell death network (37, 41). Furthermore, the components of this axis may also influence inflammatory pathways, suggesting a more integrated regulatory role that extends beyond a singular linear pathway. This concept does not exclude the contribution of classical apoptosis. Instead, it emphasizes that under severe infectious stress, multiple cell death pathways may act together on a background of metabolic collapse, contributing to extensive RPE loss. Such a framework may also help explain the clinical observation that RPE degeneration and visual impairment can continue to progress even after fungal pathogens have been effectively controlled by antifungal therapy. It is important to acknowledge that the precise temporal relationship between mitochondrial impairment and the initiation of PANoptotic signaling in the complex *in vivo* microenvironment of FE remains an area requiring further investigation. While our mechanistic model suggests that metabolic dysregulation and subsequent mitochondrial dysfunction can precede and contribute to PANoptotic activation, we also recognize that inflammatory signaling can reciprocally exacerbate mitochondrial damage, creating a complex feedback loop. Therefore, we propose that severe mitochondrial damage may act as an important amplifier of the PANoptotic response, potentially strengthening a feed-forward loop in severe inflammatory settings and contributing to persistent RPE injury and retinal damage.

### 6.3 Controversies and unresolved key questions

Although this conceptual framework provides a new perspective on FE pathogenesis, several important issues remain unresolved.

First, within the complex *in vivo* microenvironment of FE, the exact characteristics of RPE cell death require further clarification. It remains uncertain whether RPE cells undergo classical PANoptosis or instead display stage−dependent transitions between pyroptosis and necroptosis at different infection time points. Addressing this question will likely require detailed time-course studies and spatiotemporal analyses at the *in vivo* single−cell level.

Another key consideration involves receptor abundance. Basal ZBP1 expression is extremely low in normal RPE cells, raising the question of whether its expression can increase sufficiently during fungal infection and inflammatory signaling to permit PANoptosome assembly. This represents a central challenge in validating the proposed mechanism.

In addition, potential iatrogenic influences should be considered. High−dose antifungal agents frequently used in clinical practice, such as amphotericin B, may possess intrinsic cellular or mitochondrial toxicity. Whether such effects contribute to mtDNA leakage and accelerate irreversible RPE damage requires careful biochemical and pharmacological investigation.

Finally, these mechanistic insights raise an important therapeutic question: when is the optimal time window for host−directed interventions targeting metabolic homeostasis? Such interventions might be applied early alongside antifungal treatment to prevent structural collapse, or alternatively after partial pathogen control to promote tissue recovery. Clarifying this issue will be critical for designing future animal studies and clinical trials.

### 6.4 Translational prospects and biomarkers

Future work should focus on validating the relevance of this signaling axis in non−human primate models and clinical specimens while exploring biomarkers and therapeutic strategies derived from this mechanism. One promising Candidate biomarker is cell−free mtDNA in intraocular fluids such as aqueous humor or vitreous, together with its oxidative modification status. These parameters may serve as sensitive indicators of RPE mitochondrial damage and potential predictors of visual outcomes (35). Clinically, minimally invasive sampling of small volumes of intraocular fluid combined with droplet digital PCR (ddPCR) could enable precise quantification of cell−free mtDNA copy number and oxidative damage levels. For such quantitative biomarker analyses, robust statistical methods, with a P-value threshold of <0.05 (and appropriate multiple comparison corrections if assessing multiple markers), will be critical to ensure reliable distinction between disease states and prognostic stratification. Integrating these measurements with structural findings from optical coherence tomography (OCT) and functional assessments from electroretinography (ERG) may help establish prognostic stratification frameworks. In addition, molecular markers associated with the PANoptotic pathway—such as GSDMD cleavage fragments (GSDMD−N), activated Caspase−1, or phosphorylated MLKL—could be explored as indicators of inflammatory retinal injury. When conducting high-throughput screens or quantitative analyses for these markers (e.g., proteomics or multiplex assays), we anticipate employing statistical thresholds such as an adjusted *P*-value (FDR) < 0.05 and a |Fold Change| ≥ 2.0 to identify significant and biologically relevant alterations. Detection of ZBP1 phosphorylation or MITF degradation products may further support stratified therapeutic decision−making. From a therapeutic perspective, combining conventional antifungal agents with strategies aimed at preserving mitochondrial function—such as small−molecule FBXW7 inhibitors or mitochondrial protective agents—may represent a potential dual−target approach for preserving retinal function in FE. Such strategies may help shift treatment paradigms from a purely fungicidal approach toward a combined fungicidal and retinoprotective model.
